# *ATG16L1* and *ATG12* Gene Polymorphisms Are Involved in the Progression of Atrophic Gastritis

**DOI:** 10.3390/jcm12165384

**Published:** 2023-08-19

**Authors:** Naoyuki Yamaguchi, Takuki Sakaguchi, Hajime Isomoto, Tatsuo Inamine, Haruka Ueda, Daisuke Fukuda, Ken Ohnita, Tsutomu Kanda, Hiroki Kurumi, Kayoko Matsushima, Tatsuro Hirayama, Kazuo Yashima, Kazuhiro Tsukamoto

**Affiliations:** 1 Department of Gastroenterology and Hepatology, Nagasaki University Graduate School of Biological Sciences, 1-7-1 Sakamoto, Nagasaki 852-8501, Japan; 2 Department of Gastroenterology and Nephrology, Faculty of Medicine, Tottori University, 36-1 Nishi-cho, Yonago 683-8504, Japan; 3 Department of Pharmacotherapeutics, Nagasaki University Graduate School of Biomedical Sciences, 1-7-1 Sakamoto, Nagasaki 852-8501, Japan; 4 Department of Surgical Oncology, Nagasaki University Graduate School of Biological Science, 1-7-1 Sakamoto, Nagasaki 852-8501, Japan; 5 Fukuda Yutaka Clinic, 3-5 Hamaguchi-machi, Nagasaki 852-8107, Japan; 6 Shunkaikai Inoue Hospital, 6-12 Takara-machi, Nagasaki 850-0045, Japan

**Keywords:** gastric mucosal atrophy, *ATG16L1*, *ATG12*, SNPs

## Abstract

*Helicobacter pylori* (*H. pylori*) infection causes a progression to atrophic gastritis and results in gastric cancer. Cytotoxin-associated gene A (CagA), a major virulence factor of *H. pylori*, is injected into gastric epithelial cells using the type IV secretion system. On the other hand, gastric epithelial cells degrade CagA using an autophagy system, which is strictly regulated by the autophagy-related (ATG) genes. This study aimed to identify SNPs in *ATG5*, *ATG10*, *ATG12*, and *ATG16L1* associated with gastric mucosal atrophy (GMA). Here, two-hundred *H. pylori*-positive participants without gastric cancer were included. The degree of GMA was evaluated via the pepsinogen method. Twenty-five SNPs located in the four candidate genes were selected as tag SNPs. The frequency of each SNP between the GMA and the non-GMA group was evaluated. The rs6431655, rs6431659, and rs4663136 in *ATG16L1* and rs26537 in *ATG12* were independently associated with GMA. Of these four SNPs, the G/G genotype of rs6431659 in *ATG16L1* has the highest odd ratio (Odds ratio = 3.835, 95% confidence intervals = 1.337–1.005, *p* = 0.008). Further functional analyses and prospective analyses with a larger sample size are required.

## 1. Introduction

*Helicobacter pylori* (*H. pylori*), a Gram-negative bacillus, was defined as a “definite carcinogen” for gastric cancer by the International Agency for Research on Cancer, a subordinate organization of the World Health Organization, in 1994 [1,2]. Persistent infection with *H. pylori* causes atrophic gastritis and intestinal metaplasia, resulting in gastric cancer [3]. When *H. pylori* adheres to gastric epithelial cells, it injects cytotoxin-associated gene A (CagA) into the epithelial cell using a type IV secretion system (T4SS) [4]. The injected CagA undergoes tyrosine phosphorylation by Src family kinase and c-ALB kinase [5]. The tyrosine-phosphorylated CagA interacts with Src homology 2 (SHP2) [6] and enhances the Ras-ERK pathway [7]. CagA also binds to the polarity-regulating serine/threonine kinase (PAR1), and as a result, the polarity of gastric epithelial cells is destroyed [8,9]. Furthermore, CagA provokes chronic atrophic gastritis by inducing inflammatory cytokines, such as interleukin (IL)-1, IL-6, IL-8, IL-18, and tumor necrosis factor α (TNFα) [4,10]. As the degree of gastric mucosal atrophy increases, the risk of developing gastric cancer increases [11]. Thus, preventing the development of severe gastritis is essential. The relationship between *H. pylori* and the host’s autophagy is very complicated. The vacuolating toxin (VacA), another toxin that causes vacuolation and mitochondrial damage, initiates autophagy [12]. On the other hand, autophagy conversely reduces the intracellular VacA [12]. Focusing on the relationship between CagA and autophagy, the host’s autophagy degrades the CagA as a host defense mechanism [13,14].

**Figure 1:**
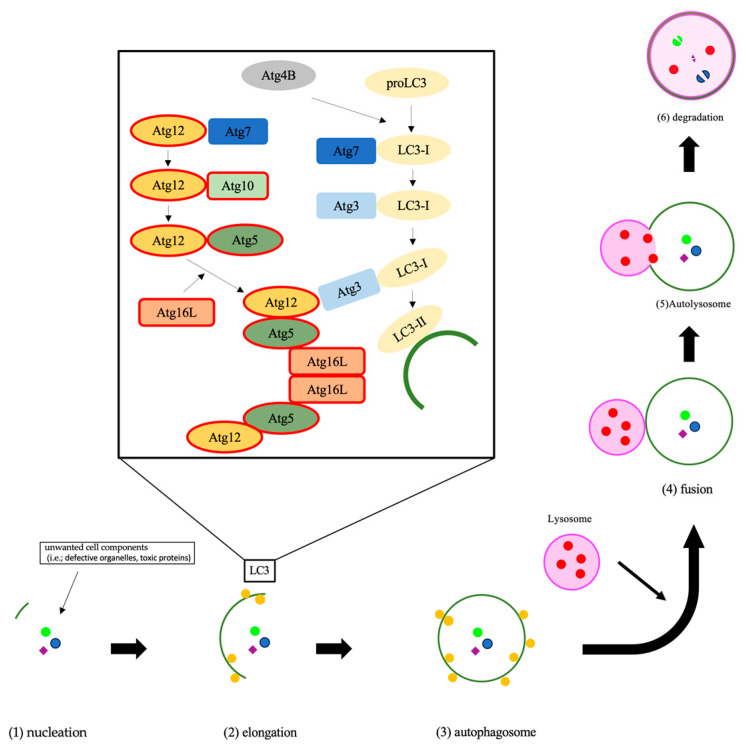
The process of autophagy is shown. The initial step of autophagy is nucleation, which is the appearance of a double membrane (1). The double membrane began to elongate and isolate the unwanted cell comportments, which are named autophagosomes (2) and (3). Then, the lysosome fuses with the autophagosome (4), which is termed an autolysosome (5), and degrades the autophagic cargo (6). The Atg12 covalent bond system contributes to autophagosome membrane elongation. The Atg12-Atg5-Atg16 complex controls microtubule-associated protein 1, light chain 3, alpha, which completes the autophagosome, to localize to the membrane. Abbreviations: LC3, microtubule-associated protein 1, light chain 3, alpha; PI3P, phosphatidylinositol (3,4,5)-trisphosphate.

Autophagy is an essential system, which is highly conserved in eukaryotes, for the degradation of unwanted cell components and subsequent recycling of cellular material to keep homeostasis and functions [15,16,17]. In the autophagy process, a lipid bilayer membrane named the phagophore appears, expands, and isolates the intra-cellular cargo with double-membraned autophagosome [18]. The phagophore fuses with the lysosome to form an autolysosome [19]. The autophagy-related (ATG) genes strictly regulate autophagic flux [19]. In these ATG proteins, ATG10 conjugates ATG12 to ATG5, and the ATG5-ATG12 complex conjugates with ATG16. The ATG5-ATG12-ATG16 complex expands the autophagosome membrane (Figure 1) [18]. On the other hand, ATG4 is also important in autophagy flux. ATG4 changes proLC3 to LC3-I, then the E1-like enzyme Atg7 and the E2-like ATG3 change LC3-I to LC3-II (Figure 1) [20]. Recent studies have revealed that autophagy is associated with various diseases [21,22,23]. In the field of cancer, autophagy can either promote or inhibit tumorigenesis or cancer cell proliferation [24,25].

It has been reported that *ATG2B*, *ATG5, ATG9B*, *ATG12*, and *ATG16L1* are closely related to gastric cancer (GC) [23]. The expressions of *ATG5* and the *ATG16L1* were downregulated in GC [26,27], and some ATG genes are also related to overall survival (OS) [23]. Furthermore, some genomic variations of ATG genes were reported as GC risk factors [25,28]. Frameshift mutation mononucleotide repeats of *ATG2B*, *ATG5*, *ATG9B*, and *ATG12* are common in GC with MSI-H [29]. The rs10205233 T-allele in *IRS1*, an autophagy-related gene involved in the PIK/Akt/mTOR pathway, decreases the risk of incidence of GC [25]. The rs2241880 G-allele in *ATG16L1* is associated with the development of GC in the Netherlands and Australia because it reduces ER stress [28]. However, the relationship between gene polymorphisms in *ATG5*, *ATG10*, *ATG12*, and *ATG16L1* and gastric mucosal atrophy (GMA) progression has yet to be comprehensively elucidated. In this study, we aimed to clarify the relationship between GMA and SNPs in *ATG5*, *ATG10*, *ATG12*, and *ATG16L1* and to explore biomarkers for the progression of GMA.

## 2. Materials and Methods

### 2.1. Study Subjects

The subjects were two hundred *H. pylori*-infected patients (*H. pylori* antibody titers ≥ 10 U/mL, E-plate Eiken *H. pylori* antibody II; Eiken Chemical, Tokyo, Japan) [30] among five hundred and three patients who underwent esophagogastroduodenoscopy at Fukuda Yutaka Clinic for their health check-up, as previously reported [31]. Participants who were younger than eighty and had no *H. pylori* eradication history were included. Clinical information of the subjects is shown in Table 1.

Written informed consent was obtained from all the subjects. The present study was approved by the Human Genome and Gene Analysis Research Ethics Committee of Nagasaki University (No. 120221, approved on 16 February 2012).

### 2.2. Classification of the Degree of Atrophy

The pepsinogen (PG) method was used to evaluate the degree of atrophy. PG has two serum types: PG I and PG II. We classified those whose PG I value was <70 µg/L (PG I < 70) and PG I/II ratio was <3.0 (PG I/II < 3.0) into a GMA group [32]. Patients not meeting those criteria were classified into a non-GMA group.

### 2.3. Genomic DNA Extraction from Peripheral Blood

DNA was extracted from blood cells in the peripheral blood of each patient using NucleoSpin^®^ (Takara, Shiga, Japan) according to the instructions. The extracted DNA concentration was measured using a Nanodrop^®^ UD-1000 (Nanodrop Technologies, Wilmington, DE, USA), and the final concentration was adjusted to 15 ng/μL by adding low TE (10 mM Tris-HCl, pH 8.0, 0.1 mM EDTA).

### 2.4. Selection of Tag SNPs of the Candidate Genes

**Figure 2:**
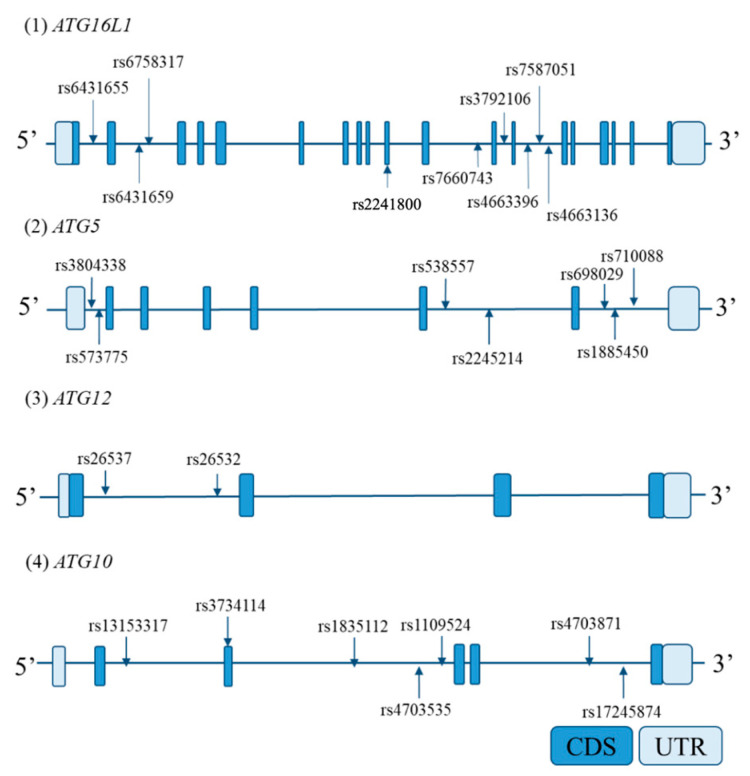
Locations of genotyped tag single-nucleotide polymorphisms (SNPs) in each gene. The horizontal bars indicate the genomic sequence of each candidate gene. The boxes on the bars represent exons. Arrows indicate the genotyped tag SNP sites. Abbreviations: CDS, coding sequence; UTR, untranslated region.

Based on the International HapMap database, all SNPs reported in Japanese that were in *ATG5*, *ATG10*, *ATG12*, and *ATG16L1* and a region up to 2 kb upstream from each candidate gene promoter were extracted. From the extracted SNPs, those with a minor allele frequency of ≥0.1 were selected; then, tag SNPs were selected using a pair-wise tagging method using Haploview 4.2 software [33] (r^2^ > 0.8). Locations of tag SNPs in each candidate gene are shown in Figure 2.

### 2.5. Polymorphism Analysis

The selected tag SNPs were genotyped using a PCR-restriction fragment length polymorphism method, a PCR-direct DNA sequencing method, or a PCR-High-Resolution Melting (HRM) analysis with a nonlabelled probe method. Primers for polymerase chain reaction (PCR) were designed to amplify a fragment containing each tag SNP. Table 2 shows the primer sequences, annealing temperatures, number of cycles, and typing methods (including restriction enzymes).

#### 2.5.1. PCR-Restriction Fragment Length Polymorphism Method

Each polymorphic region was amplified via PCR method with a GeneAmp PCR System 9700 (Life Technologies, Carlsbad, CA, USA) or T100 Thermal Cycler (Bio-Rad, Hercules, CA, USA). The PCR solution was composed of 10 ng of genomic DNA, 1 × Go Taq^®^ Green Mater Mix (Promega, Madison, WI, USA) and 0.6 µM each of forward and reverse primers in a total volume of 20 µL. After denaturation at 95 °C for 2 min, a cycle reaction (at 95 °C for 30 s, at the annealing temperature specific to each primer set for 30 s, and at 72 °C for 30 s for extension) was performed for the number of times specific to each primer set, and extension was performed at 72 °C for 5 min at the end (Supplementary Table S1).

After amplification, PCR products were separated via electrophoresis with 2% ME agarose gel (Nacalai Tesque, Tokyo, Japan), stained with ethidium bromide, and then detected under UV illumination.

Subsequently, PCR products were digested with restriction enzymes (Supplementary Table S1) and were separated via electrophoresis with 2% ME agarose gel. After electrophoresis ended, bands were detected, and genotypes were determined.

#### 2.5.2. PCR-Direct DNA Sequencing Method

Each polymorphic region was amplified via PCR method with the same reaction solution composition and conditions used for the PCR-restriction fragment length polymorphism method. After PCR, 1 µL of ExoSAP-IT (GE Healthcare, Little Chalfont, UK) was added to 5 µL of PCR product to inactivate dNTPs and PCR primers, and the mixture was kept at 37 °C for 20 min for an enzymatic reaction. Then, the mixed solutions were incubated at 80 °C for 20 min to inactivate enzymes. Subsequently, a cycle sequencing reaction was performed according to the protocol of the BigDye^®^ Terminator v3.1 Cycle Sequencing Kit (Life Technologies). The reaction solution contained 25 ng of template DNA, 5× sequencing buffer, BigDye Terminator v3, and forward primer 0.1 µM or reverse primer 0.1 µM, and its total volume was adjusted to 10 µL with distilled water. The cycle sequencing reaction was incubated at 96 °C for 30 s, then carried out for 25 cycles (at 96 °C for 10 s, at 50 °C for 5 s, and at 60 °C for 4 min), and was finally performed at 60 °C for 4 min for extension. The reaction solution was purified using a Sephadex G-50 superfine column (GE Healthcare). After the reaction product was dried, 15 µL of Hi-Di formamide (Life Technologies) was added. Subsequently, the solutions were incubated at 95 °C for 2 min and left on ice for at least 5 min. The DNA sequence was determined by performing capillary electrophoresis using an ABI PRISM 3100-Avant Genetic Analyzer (Life Technologies).

#### 2.5.3. PCR-HRM Analysis with a Nonlabelled Probe

Each polymorphic region was amplified via the PCR method. The PCR reaction solution was composed of 10 ng of genomic DNA, 1× Go Taq^®^ Colorless Mater Mix, 0.06 µM forward primer, 0.3 µM reverse primer, 0.3 µM probe, and 2 µM SYTO9 (Life Technologies) in a total volume of 20 µL (Supplementary Tables S1 and S2). Probes were 25–30 base oligonucleotides with complementary sequences to major alleles of tag SNPs. The 3′ ends of the probes were modified to prevent the extension of themselves.

The HRM reaction of each PCR product was performed using a LightCycler 480 Instrument (Roche Diagnostics, Basel, Switzerland). Regarding the HRM condition, each PCR product was heat-denatured at 95 °C for 1 min and then renatured at 40 °C for 1 min. After that, a change in the amount of fluorescence as the temperature changed from 50 °C to 95 °C was captured. Using the LightCycler 480 Gene-Scanning software version 1.5, the melting curves of probes were analyzed to determine genotypes. We analyzed the arbitrarily selected samples using PCR-direct DNA sequencing to check the accuracy.

### 2.6. Statistical Analysis

The Mann–Whitney *U*-test or chi-squared test was used to compare clinical information between the GMA and non-GMA groups. In polymorphism analysis, the chi-squared test evaluated whether each SNP met the Hardy–Weinberg equilibrium (HWE). For SNPs that met the HWE, the frequencies of alleles and genotypes were compared in three genetic models (allele, minor allele dominant, and minor allele recessive) using the chi-squared or Fisher’s exact test. The SNPs that showed significant differences with chi-squared or Fisher’s exact test were subjected to multivariate logistic regression analysis to verify independence between age and genotype. A value of *p* < 0.05 was considered to indicate a significant difference. The SNPAlyze 7.0 (Dynacom Co., Ltd., Yokohama, Japan), IBM SPSS Statistics 20 software package (IBM Japan, Tokyo, Japan), or Prism 6 (GraphPad Software, Inc., La Jolla, CA, USA) was used to calculate the odds ratios (ORs) and 95% confidence intervals (CIs).

## 3. Results

### 3.1. Comparison of Clinical Information

The clinical information of the GMA and non-GMA groups is shown in Table 1. The mean age in the non-GMA group was younger than that in the GMA group (54.8 ± 10.92 vs. 59.1 ± 9.51, *p* = 0.002). There was no difference between those two groups in sex (*p* = 0.266) (Table 1).

### 3.2. Analysis of the Correlation between SNPs in ATG5, ATG10, ATG12, and ATG16L1 and GMA

First, we conducted the HWE test. As a result, rs4663396 in *ATG16L1* did not meet the HWE and was excluded from additional SNP analyses. All other SNPs in the ATG-related genes met the HWE.

Next, we analyzed the association between the SNPs in four ATG genes and GMA using an allele model, a minor allele dominant model, and a minor allele recessive model. The results of the SNP analyses are shown in Table 2. Multivariate logistic regression analyses between each genetic model of each SNP and age were conducted. The *p* values of the multivariate logistic regression analysis are shown as a correction *p* value.

The frequencies of the dominant model (A/G or G/G genotype) and the recessive model (G/G genotype) of rs6431655 in *ATG16L1* were significantly higher in the GMA group compared to those in the non-GMA group (dominant model; *p* = 0.024, OR = 1.951, 95%CI = 1.087–3.500, recessive model; *p* = 0.005, OR = 2.838, 95%CI = 1.334–6.040). In addition, the recessive model of rs6431659 (G/G genotype), of rs7587051 (C/C genotype), and of rs4663136 and the dominant model of rs4663136 in *ATG16L1* were associated with the GMA (rs6431659; *p* = 0.008, OR = 3.835, 95%CI = 1.337–11.005, rs7587051; *p* = 0.044, OR = 2.038, 95%CI = 1.011–4.110, rs4663136 (recessive model); *p* = 0.044, OR = 2.380, 95%CI = 1.005–5.632, rs4663136 (dominant model); *p* = 0.017, OR = 1.994, 95%CI = 1.128–3.524). However, the recessive model of rs7587051 and rs4663136 did not independently contribute to GMA according to multivariate analysis (*p* = 0.058, *p* = 0.067).

On the other hand, the frequency of the recessive model (C/C genotype) of *ATG12* rs26537 was significantly lower in the GMA group compared to that in the non-GMA group (*p* = 0.033, OR = 0.357, 95%CI = 0.135–0.948). Conversely, the T/T or T/C genotype in rs26537 in *ATG12* was approximately 2.9 times more sensitive to non-GMA. The other SNPs were not associated with GMA.

Subsequently, we conducted a multivariate logistic regression analysis with a G/G genotype of rs6431659 in *ATG16L1*, T/T or T/C genotype of rs26537 in *ATG12*, and age (≥58 years). Table 3 shows the OR with 95% CI and *p* value. These three factors were independently involved in the progression of GMA.

### 3.3. Biomarkers for Indicating GMA

Table 4 shows the odds ratio, sensitivity, specificity, positive predictive value (PPV), and negative predictive value (NPV) of rs6431659 in ATG16L1, rs26537 in *ATG12*, and the combination of two SNPs for predicting GMA progression. The *ATG16L1* rs6431659 G/G genotype had the highest OR (OS = 3.835, *p* = 0.008, 95%C.I. = 1.336–11.01). Its sensitivity was 16.0%, its specificity was 95.3%, its PPV was 75.0%, and its NPV was 56.1%.

## 4. Discussion

*H. pylori* is undoubtedly a significant cause of GMA and gastric cancer. One of the main pathogeneses is CagA, which not only activates and translocates NF-κB into the nucleus but also down-regulates autophagy, leading to severe inflammation [34,35]. On the other hand, CagA is degraded by autophagy as a host defense [8]. The primary eradication therapy for *H. pylori* is a triple-drug therapy using two antibiotics and a proton pump inhibitor, which fails to eradicate the pathogen in approximately 10–30% of patients [36]. The main reasons proposed for this eradication failure are antibiotic resistance, high bacterial load, low compliance to therapy, and high gastric acidity [37]. It was also reported that the intracellular invasion of *H. pylori* is one of the causes of eradication failure [37]. Patients allergic to those antibiotics could not use triple-drug therapy. Moreover, some patients fail to continue the *H. pylori* eradication therapy because they suffer from adverse events [38]. Therefore, it is crucial to develop novel therapeutic drugs based on autophagic mechanisms that could eradicate *H. pylori* and suppress the progression of GMA.

ATG genes are associated with GC. Vigen. R.A. et al. reported that *ATG5* and *ATG16L1* were negative in eight of ten adenocarcinoma patients, whereas they were positive all in normal tissue [26]. Furthermore, An. C.H. et al. reported that *ATG5* protein was lost in 21% of the GC [27]. *ATG5* and *ATG16L1* were also associated with chemotherapy sensitivity [39,40]. Moreover, *ATG16L1* and *ATG5* mRNA levels in *H. pylori* positive is reduced compared to non-*H. pylori* infection in Bhutanese volunteers [41].

The present study suggested, for the first time, comprehensively, the A/G or G/G genotype of rs6431655 in *ATG16L1*, the G/G genotype of rs6431659 in *ATG16L1*, and the C/G or G/G genotype of rs4663136 in *ATG16L1* were susceptibility genes for GMA with *H. pylori* infection. Conversely, the C/C genotype of rs26537 in *ATG12* is a non-susceptible gene to GMA. Furthermore, multivariate logistic regression analyses revealed that the G/G genotype of rs6431659 in *ATG16L1*, T/T or T/C genotype of rs26537 in *ATG12*, and age (≤58 years) were independently involved in the progression of GMA.

Although there is no report or functional research about rs6431655, rs6431659, and rs4663136 in *ATG16L1*, it is known that these SNPs are in the introns and, therefore, may down-regulate the *ATG16L1* mRNA level and suppress autophagy via the modification of regulating sequences like an enhancer, not by changing protein structure.

The G/G genotype of rs2241880 in *ATG16L1* was previously reported to be associated with G/C in Australian volunteers and *H. pylori* infections in Bhutan volunteers [28,41]. On the other hand, the A allele of rs2241880 in *ATG16L1* is a risk factor for mild intestinal metaplasia (IM), defined as Operative Ling on Gastric Intestinal Metaplasia (OLGIM) stage I or II, in the Dutch population but not a risk factor for severe IM, defined as OLGIM stage III or IV [28]. However, in this study, rs2241880 in *ATG16L1* was not selected as a tag SNPs.

It was reported that the C/C genotype of rs26537 in *ATG12* upregulated *ATG12* mRNA levels in head and neck squamous cell carcinoma in Chinese Han populations [15]. Moreover, in HCC, the expression of *ATG12* was upregulated, and the C allele of the rs26537 in *ATG12* was increased [42]. These factors may indicate that the C allele of the rs26537 causes the upregulation of *ATG12*. Therefore, the C/C allele of rs26537 in *ATG12* might increase the *ATG12* mRNA level and enhance autophagy.

This study had several limitations. First, the sample size in this study was too small. Further prospective studies with larger sample sizes are required. Second, functional analysis of rs6431655, rs6431659, and rs4663136 in *ATG16L1* and rs26537 in *ATG12* polymorphisms was not performed. Third, we diagnosed the participants with *H. pylori* only by antibody titer of 10 U/mL or higher using Eiken E plate II. Fourth, we did not examine *H. pylori* staining and bacterial virulence factors, such as CagA and vacuolating cytotoxin A (VacA). It was also reported that VacA decreases the function of autophagy. Moreover, we did not analyze the autophagic activity levels by measuring LC3, ATG16L1, ATG12, and p62. In addition, the GMA group is older than the non-GMA group. It was reported that the PGI and PGI/PGII ratios were affected by age in a healthy Chinese population [43]. However, the multivariate logistic regression analysis reveals the G/G genotype of rs6431659 in *ATG16L1* and T/T or T/C genotype of rs26537 in *ATG12* were independently involved in the progression of GMA. We hope to conduct future exploratory studies with younger participants to equalize the age of the two groups.

## 5. Conclusions

The present study shows that *ATG16L1* and *ATG12* are susceptibility genes of *H. pylori*-infected gastritis. Although functional analysis and verification with larger sample studies are required, this study shed light on the future directions of novel therapeutic drugs based on autophagic mechanisms to prevent the progression of GMA.

## Figures and Tables

**Table 1:** Clinical characteristics of H. pylori-positive subjects.

Characteristics	GMA	Non-GMA	*p* Value
Number of subjects	94	106	-
Age, mean ± SD (years)	59.2 ± 9.52	54.9 ± 10.93	0.002
Gender (male/female)	37/57	50/56	0.266

The Mann–Whitney *U* test and chi-square test were applied to statistical analysis. Abbreviations: SD, standard deviation. Abbreviations: GMA, gastric mucosal atrophy.

**Table 2:** Frequencies of genotypes of tag SNPs in the GMA and non-GMA groups.

Gene	SNP	Genotype	Number of		Genetic Model	OR (95% CI)	*p* Value *	Correction *p* Value **
			GMA *n* = 94 (%)	non-GMA *n* = 106 (%)				
*ATG16L1*	rs6431655	A/A	28 (29.8)	48 (45.3)	Allele model	1.903 (1.270–2.852)	0.002	–
		A/G	41 (43.6)	46 (43.4)	Dominant model	1.951 (1.087–3.500)	0.024	0.027
		G/G	25 (26.6)	12 (11.3)	Recessive model	2.838 (1.334–6.040)	0.005	0.007
	rs6431659	A/A	41 (43.6)	58 (54.7)	Allele model	1.700 (1.106–2.614)	0.015	–
		A/G	38 (40.4)	43 (40.6)	Dominant model	1.562 (0.893–2.732)	0.117	–
		G/G	15 (16.0)	5 (4.7)	Recessive model	3.835 (1.337–11.005)	0.008	0.014
	rs6758317	C/C	64 (68.1)	84 (79.2)	Allele model	1.855 (1.061–3.245)	0.029	–
		C/T	24 (25.5)	20 (18.9)	Dominant model	1.790 (0.945–3.391)	0.073	–
		T/T	6 (6.4)	2 (1.9)	Recessive model	3.546 (0.698–18.011)	0.151	–
	rs2241800	T/T	57 (67.9)	72 (60.6)	Allele model	1.364 (0.828–2.245)	0.222	–
		T/C	33 (35.1)	32 (30.2)	Dominant model	1.375 (0.769–2.458)	0.283	–
		C/C	4 (4.3)	2 (1.9)	Recessive model	2.311 (0.414–12.916)	0.423	–
	rs7600743	A/A	73 (77.7)	89 (84.0)	Allele model	1.346 (0.704–2.574)	0.367	–
		A/G	20 (21.3)	15 (14.2)	Dominant model	1.506 (0.740–3.065)	0.257	–
		G/G	1 (1.1)	2 (1.9)	Recessive model	0.559 (0.050–6.268)	1.000	–
	rs3792106	A/A	46 (48.9)	66 (62.3)	Allele model	1.454 (0.923–2.291)	0.105	–
		A/G	42 (44.7)	34 (32.1)	Dominant model	1.722 (0.980–3.026)	0.058	–
		G/G	6 (6.4)	6 (5.7)	Recessive model	1.136 (0.354–3.652)	0.830	–
	rs7587051	G/G	26 (27.7)	41 (38.7)	Allele model	1.583 (1.063–2.358)	0.023	–
		G/C	43 (45.7)	49 (46.2)	Dominant model	1.650 (0.908–2.999)	0.099	–
		C/C	25 (26.6)	16 (15.1)	Recessive model	2.038 (1.011–4.110)	0.044	0.058
	rs4663136	C/C	33 (35.1)	55 (51.9)	Allele model	1.796 (1.184–2.725)	0.006	–
		C/G	44 (46.8)	42 (39.6)	Dominant model	1.994 (1.128–3.524)	0.017	0.018
		G/G	17 (18.1)	9 (8.5)	Recessive model	2.380 (1.005–5.632)	0.044	0.067
*ATG5*	rs3804338	C/C	71 (75.5)	85 (80.2)	Allele model	1.260 (0.689–2.305)	0.452	–
		C/T	21 (22.3)	19 (17.9)	Dominant model	1.311 (0.671–2.563)	0.428	–
		T/T	2 (2.1)	2 (1.9)	Recessive model	1.130 (0.156–8.187)	1.000	–
	rs573775	C/C	37 (39.4)	39 (36.8)	Allele model	1.292 (0.860–1.940)	0.217	–
		C/T	49 (52.1)	48 (45.3)	Dominant model	0.897 (0.506–1.589)	0.709	–
		T/T	8 (8.5)	19 (17.9)	Recessive model	0.426 (0.177–1.025)	0.052	–
	rs538557	T/T	51 (54.3)	58 (54.7)	Allele model	1.190 (0.716–1.863)	0.446	–
		T/C	40 (42.6)	37 (34.9)	Dominant model	1.019 (0.583–1.779)	0.948	–
		C/C	3 (3.2)	11 (10.4)	Recessive model	0.285 (0.077–1.054)	0.047	–
	rs2245214	C/C	23 (24.5)	31 (29.2)	Allele model	1.104 (0.746–1.636)	0.621	–
		C/G	46 (48.9)	47 (44.3)	Dominant model	1.276 (0.680–2.395)	0.448	–
		G/G	25 (26.6)	28 (26.4)	Recessive model	1.009 (0.538–1.893)	0.977	–
	rs698029	G/G	32 (34.0)	41 (38.7)	Allele model	1.062 (0.713–1.582)	0.768	–
		G/A	45 (47.9)	44 (41.5)	Dominant model	1.222 (0.685 –2.180)	0.497	–
		A/A	17 (18.1)	21 (19.8)	Recessive model	0.894 (0.439–1.818)	0.756	–
	rs1885450	T/T	66 (70.2)	74 (69.8)	Allele model	0.904 (0.537–1.523)	0.705	–
		T/C	25 (26.6)	26 (24.5)	Dominant model	0.981 (0.535–1.799)	0.951	–
		C/C	3 (3.2)	6 (5.7)	Recessive model	0.550 (0.134–2.261)	0.505	–
	rs10088	T/T	53 (56.4)	58 (54.7)	Allele model	1.262 (0.803–1.983)	0.312	–
		T/C	38 (40.4)	37 (34.9)	Dominant model	0.935 (0.535–1.635)	0.813	–
		C/C	3 (3.2)	11 (10.4)	Recessive model	0.285 (0.077–1.054)	0.047	–
*ATG12*	rs26537	T/T	38 (40.4)	40 (37.7)	Allele model	0.765 (0.507–1.153)	0.200	–
		T/C	50 (53.2)	49 (46.2)	Dominant model	0.893 (0.506–1.578)	0.697	–
		C/C	6 (6.4)	17 (16.0)	Recessive model	0.357 (0.135–0.948)	0.033	0.012
	rs26532	A/A	29 (30.9)	38 (35.8)	Allele model	1.358 (0.909–2.028)	0.134	–
		A/C	47 (50.0)	58 (54.7)	Dominant model	1.253 (0.694–2.262)	0.455	–
		C/C	18 (19.1)	10 (9.4)	Recessive model	2.274 (0.992–5.212)	0.048	–
*ATG10*	rs13153317	A/A	37 (39.4)	52 (49.1)	Allele model	1.343 (0.889–2.030)	0.161	–
		A/C	42 (44.7)	41 (38.7)	Dominant model	1.484 (0.845–2.603)	0.169	–
		C/C	15 (16.0)	13 (12.3)	Recessive model	1.358 (0.610–3.026)	0.453	–
	rs3734114	T/T	74 (78.7)	87 (82.1)	Allele model	1.144 (0.603–2.168)	0.681	–
		T/C	19 (20.2)	17 (16.0)	Dominant model	1.238 (0.614–2.493)	0.550	–
		C/C	1 (1.1)	2 (1.9)	Recessive model	0.559 (0.050–6.268)	1.000	–
	rs1835112	T/T	39 (41.5)	36 (34.0)	Allele model	0.861 (0.577–1.284)	0.462	–
		T/G	37 (39.4)	50 (47.2)	Dominant model	0.725 (0.408–1.288)	0.273	–
		G/G	18 (19.1)	20 (18.9)	Recessive model	1.018 (0.502–2.067)	0.960	–
	rs4703535	A/A	71 (75.5)	83 (78.3)	Allele model	1.047 (0.579–1.895)	0.880	–
		A/T	22 (23.4)	20 (18.9)	Dominant model	1.169 (0.605–2.260)	0.642	–
		T/T	1 (1.1)	3 (2.8)	Recessive model	0.369 (0.038–3.611)	0.624	–
	rs1109524	T/T	30 (31.9)	29 (27.4)	Allele model	0.782 (0.527–1.159)	0.220	–
		T/C	42 (44.7)	44 (41.5)	Dominant model	0.804 (0.437–1.477)	0.481	–
		C/C	22 (23.4)	33 (31.3)	Recessive model	0.676 (0.360–1.269)	0.222	–
	rs4703871	C/C	72 (76.6)	85 (80.2)	Allele model	1.092 (0.594–2.008)	0.777	–
		C/T	21 (22.3)	18 (17.0)	Dominant model	1.237 (0.630–2.430)	0.537	–
		T/T	1 (1.1)	3 (2.8)	Recessive model	0.369 (0.038–3.611)	0.624	–
	rs17245874	C/C	38 (40.4)	29 (27.4)	Allele model	0.762 (0.513–1.133)	0.179	–
		C/T	35 (37.2)	53 (50.0)	Dominant model	0.555 (0.307–1.005)	0.051	–
		T/T	21 (22.3)	24 (22.6)	Recessive model	0.983 (0.505–1.912)	0.959	–

* Alleles and genotypes in three genetic models were compared using chi-square or Fisher’s exact test. ** Genotype and age (≥58) were statistically analyzed via multivariate logistic regression analysis. Abbreviation: SNP, single-nucleotide polymorphism; OR, odds ratio; CI, confidence interval.

**Table 3:** Multivariate logistic regression analysis of rs6431659 in *ATG16L1*, rs26537 in *ATG12*, and age.

Factor	OR (95% CI)	*p* Value *
G/G genotype of rs6431659 in *ATG16L1*	3.579 (1.216–10.532)	0.021
T/T or T/C genotype of rs26537 in *ATG12*	3.466 (1.244–9.659)	0.017
Age (≥58)	2.570 (1.414–4.672)	0.002

* Factors were statistically analyzed using multivariate logistic regression analysis.

**Table 4:** The sensitivity, specificity, PPV, and NPV of the G/G genotype of rs6431659 in *ATG16L1* and/or the T/T or T/C genotype of rs26537 in *ATG12* as a biomarker of GMA progression.

Biomarker	*ATG16L1*	*ATG12*	Statistical Results		Sensitivity	Specificity	PPV	NPV
	rs6431659	rs26537	OR (95% CI)	*p* Value *				
marker1	G/G	-	3.835 (1.336–11.01)	0.008	16.0	95.3	75.0	56.1
marker2	-	T/T or T/C	2.801 (1.055–7.438)	0.033	93.6	16.0	49.7	73.9
marker2	G/G	T/T or T/C	3.535 (1.221–10.23)	0.014	14.9	95.3	73.7	55.8

* The chi-square test was conducted for the calculated *p* value. Abbreviation: OR, odds ratio; CI, confidence interval; PPV, positive predictive value; NPV, negative predictive value.

## Data Availability

The datasets generated during the current study are not publicly available due to data sharing not being written in the informed consent.

## Supplementary Materials

The following supporting information can be downloaded at: https://www.mdpi.com/article/10.3390/jcm12165384/s1. Table S1: This table shows the sequencing primer, annealing temperate, cycle number, analytical method, and restriction enzyme of each SNP. Table S2: This table shows the sequence of the probes and the melting temperate of each SNP.

## References

[1] Herrero R., Heise K., Acevedo J., Cook P., Gonzalez C., Gahona J., Cortés R., Collado L., Beltrán M.E., Cikutovic M. (2020). Regional variations in *Helicobacter pylori* infection, gastric atrophy and gastric cancer risk: The ENIGMA study in Chile. PLoS ONE.

[2] Moss S.F. (2017). The Clinical Evidence Linking *Helicobacter pylori* to Gastric Cancer. Cell. Mol. Gastroenterol. Hepatol..

[3] Lahner E., Conti L., Annibale B., Corleto V.D. (2020). Current Perspectives in Atrophic Gastritis. Curr. Gastroenterol. Rep..

[4] Hatakeyama M. (2017). Structure and function of *Helicobacter pylori* CagA, the first-identified bacterial protein involved in human cancer. Proc. Jpn. Acad. Ser. B Phys. Biol. Sci..

[5] Imai S., Ooki T., Murata-Kamiya N., Komura D., Tahmina K., Wu W., Takahashi-Kanemitsu A., Knight C.T., Kunita A., Suzuki N. (2021). *Helicobacter pylori* CagA elicits BRCAness to induce genome instability that may underlie bacterial gastric carcinogenesis. Cell Host Microbe.

[6] Hatakeyama M. (2004). Oncogenic mechanisms of the *Helicobacter pylori* CagA protein. Nat. Rev. Cancer.

[7] Murata-Kamiya N., Hatakeyama M. (2022). *Helicobacter pylori*-induced DNA double-stranded break in the development of gastric cancer. Cancer Sci..

[8] Tsugawa H., Suzuki H., Saya H., Hatakeyama M., Hirayama T., Hirata K., Nagano O., Matsuzaki J., Hibi T. (2012). Reactive oxygen species-induced autophagic degradation of *Helicobacter pylori* CagA is specifically suppressed in cancer stem-like cells. Cell Host Microbe.

[9] Saadat I., Higashi H., Obuse C., Umeda M., Murata-Kamiya N., Saito Y., Lu H., Ohnishi N., Azuma T., Suzuki A. (2007). *Helicobacter pylori* CagA targets PAR1/MARK kinase to disrupt epithelial cell polarity. Nature.

[10] Yamauchi K., Choi I.J., Lu H., Ogiwara H., Graham D.Y., Yamaoka Y. (2008). Regulation of IL-18 in *Helicobacter pylori* infection. J. Immunol..

[11] Masuyama H., Yoshitake N., Sasai T., Nakamura T., Masuyama A., Zuiki T., Kurashina K., Mieda M., Sunada K., Yamamoto H. (2015). Relationship between the degree of endoscopic atrophy of the gastric mucosa and carcinogenic risk. Digestion.

[12] Terebiznik M.R., Raju D., Vázquez C.L., Torbricki K., Kulkarni R., Blanke S.R., Yoshimori T., Colombo M.I., Jones N.L. (2009). Effect of *Helicobacter pylori*’s vacuolating cytotoxin on the autophagy pathway in gastric epithelial cells. Autophagy.

[13] Takahashi-Kanemitsu A., Knight C.T., Hatakeyama M. (2020). Molecular anatomy and pathogenic actions of *Helicobacter pylori* CagA that underpin gastric carcinogenesis. Cell Mol. Immunol..

[14] Nakamura K., Urabe Y., Kagemoto K., Yuge R., Hayashi R., Ono A., Hayes C.N., Oka S., Ito M., Nishisaka T. (2020). Genomic Characterization of Non-Invasive Differentiated-Type Gastric Cancer in the Japanese Population. Cancers.

[15] Song X., Yuan Z., Yuan H., Wang L., Ji P., Jin G., Dai J., Ma H. (2018). ATG12 expression quantitative trait loci associated with head and neck squamous cell carcinoma risk in a Chinese Han population. Mol. Carcinog..

[16] Rehman N.U., Zeng P., Mo Z., Guo S., Liu Y., Huang Y., Xie Q. (2021). Conserved and Diversified Mechanism of Autophagy between Plants and Animals upon Various Stresses. Antioxidants.

[17] Vijayakumar K., Cho G.W. (2019). Autophagy: An evolutionarily conserved process in the maintenance of stem cells and aging. Cell Biochem. Funct..

[18] Glick D., Barth S., Macleod K.F. (2010). Autophagy: Cellular and molecular mechanisms. J. Pathol..

[19] Levy J.M.M., Towers C.G., Thorburn A. (2017). Targeting autophagy in cancer. Nat. Rev. Cancer.

[20] Zhang L., Li J., Ouyang L., Liu B., Cheng Y. (2016). Unraveling the roles of Atg4 proteases from autophagy modulation to targeted cancer therapy. Cancer Lett..

[21] Klionsky D.J., Petroni G., Amaravadi R.K., Baehrecke E.H., Ballabio A., Boya P., Bravo-San Pedro J.M., Cadwell K., Cecconi F., Choi A.M.K. (2021). Autophagy in major human diseases. Embo J..

[22] Levine B., Kroemer G. (2008). Autophagy in the pathogenesis of disease. Cell.

[23] Wu M., Chen B., Pan X., Su J. (2020). Prognostic Value of Autophagy-related Proteins in Human Gastric Cancer. Cancer Manag. Res..

[24] White E. (2015). The role for autophagy in cancer. J. Clin. Investig..

[25] Zheng W., Wu C., Wu X., Cai Y., Liu B., Wang C. (2021). Genetic variants of autophagy-related genes in the PI3K/Akt/mTOR pathway and risk of gastric cancer in the Chinese population. Gene.

[26] Vigen R.A., Kodama Y., Viset T., Fossmark R., Waldum H., Kidd M., Wang T.C., Modlin I.M., Chen D., Zhao C.M. (2013). Immunohistochemical evidence for an impairment of autophagy in tumorigenesis of gastric carcinoids and adenocarcinomas in rodent models and patients. Histol. Histopathol..

[27] An C.H., Kim M.S., Yoo N.J., Park S.W., Lee S.H. (2011). Mutational and expressional analyses of ATG5, an autophagy-related gene, in gastrointestinal cancers. Pathol. Res. Pract..

[28] Mommersteeg M.C., Simovic I., Yu B., van Nieuwenburg S.A.V., Bruno I.M.J., Doukas M., Kuipers E.J., Spaander M.C.W., Peppelenbosch M.P., Castaño-Rodríguez N. (2022). Autophagy mediates ER stress and inflammation in *Helicobacter pylori*-related gastric cancer. Gut Microbes.

[29] Kang M.R., Kim M.S., Oh J.E., Kim Y.R., Song S.Y., Kim S.S., Ahn C.H., Yoo N.J., Lee S.H. (2009). Frameshift mutations of autophagy-related genes ATG2B, ATG5, ATG9B and ATG12 in gastric and colorectal cancers with microsatellite instability. J. Pathol..

[30] Toyoshima O., Nishizawa T., Sakitani K., Yamakawa T., Takahashi Y., Yamamichi N., Hata K., Seto Y., Koike K., Watanabe H. (2018). Serum anti-*Helicobacter pylori* antibody titer and its association with gastric nodularity, atrophy, and age: A cross-sectional study. World J. Gastroenterol..

[31] Yamaguchi N., Sakaguchi T., Isomoto H., Inamine T., Tsukamoto R., Fukuda D., Ohnita K., Kanda T., Matsushima K., Hirayama T. (2023). Polymorphism in autophagy-related genes LRP1 and CAPZA1 may promote gastric mucosal atrophy. Genes Environ..

[32] Cha J.H., Jang J.S. (2020). Clinical correlation between serum pepsinogen level and gastric atrophy in gastric neoplasm. Korean J. Intern. Med..

[33] Barrett J.C., Fry B., Maller J., Daly M.J. (2005). Haploview: Analysis and visualization of LD and haplotype maps. Bioinformatics.

[34] Li N., Tang B., Jia Y.P., Zhu P., Zhuang Y., Fang Y., Li Q., Wang K., Zhang W.J., Guo G. (2017). *Helicobacter pylori* CagA Protein Negatively Regulates Autophagy and Promotes Inflammatory Response via c-Met-PI3K/Akt-mTOR Signaling Pathway. Front. Cell Infect. Microbiol..

[35] Brandt S., Kwok T., Hartig R., König W., Backert S. (2005). NF-kappaB activation and potentiation of proinflammatory responses by the *Helicobacter pylori* CagA protein. Proc. Natl. Acad. Sci. USA.

[36] Sugimoto M., Yamaoka Y. (2018). Role of Vonoprazan in *Helicobacter pylori* Eradication Therapy in Japan. Front. Pharmacol..

[37] Wang Y.H., Lv Z.F., Zhong Y., Liu D.S., Chen S.P., Xie Y. (2017). The internalization of *Helicobacter pylori* plays a role in the failure of *H. pylori* eradication. Helicobacter.

[38] Hafeez M., Qureshi Z.A., Khattak A.L., Saeed F., Asghar A., Azam K., Khan M.A. (2021). *Helicobacter pylori* Eradication Therapy: Still a Challenge. Cureus.

[39] Huang H., Tang J., Zhang L., Bu Y., Zhang X. (2018). miR-874 regulates multiple-drug resistance in gastric cancer by targeting ATG16L1. Int. J. Oncol..

[40] Ge J., Chen Z., Huang J., Chen J., Yuan W., Deng Z., Chen Z. (2014). Upregulation of autophagy-related gene-5 (ATG-5) is associated with chemoresistance in human gastric cancer. PLoS ONE.

[41] Tanaka S., Nagashima H., Uotani T., Graham D.Y., Yamaoka Y. (2017). Autophagy-related genes in *Helicobacter pylori* infection. Helicobacter.

[42] Shen M., Lin L. (2019). Functional variants of autophagy-related genes are associated with the development of hepatocellular carcinoma. Life Sci..

[43] Shan J.H., Bai X.J., Han L.L., Yuan Y., Sun X.F. (2017). Changes with aging in gastric biomarkers levels and in biochemical factors associated with *Helicobacter pylori* infection in asymptomatic Chinese population. World J. Gastroenterol..

